# The Role of the Insulin-Like Growth Factors and Their Binding Proteins in Glucose Homeostasis

**DOI:** 10.1155/EDR.2003.213

**Published:** 2003

**Authors:** Liam J. Murphy

**Affiliations:** 1 Departments of Internal Medicine and Physiology University of Manitoba Winnipeg Manitoba Canada; 2 Room 843 John Buhler Research Centre University of Manitoba 715 McDermot Avenue Winnipeg Manitoba R3E 3P4 Canada

## Abstract

The insulin like growth factors (IGF-I and -II) are structurally and functionally related to insulin. While insulin is a key regulator of glucose homeostasis over the short term, emerging evidence suggests that the IGFs are involved in the longer term glucose homeostasis, possibly by modulating insulin sensitivity. Unlike insulin, the IGFs are present in most biological fluids as complexes with high affinity binding proteins, the insulin-like growth factor binding proteins (IGFBPs). The IGFBPs regulate the bioavailability of the IGFs. Of the six IGFBPs identified there is evidence from studies in transgenic mice that both IGFBP-1 and IGFBP-3 may have a role in glucose regulation.


*Experimental Diab. Res.*, 4:213–224, 2003
Copyright © Taylor and Francis Inc.
ISSN: 1543-8600 print / 1543-8619 online
DOI: 10.1080/15438600390249673

### The Role of the Insulin-Like Growth Factors and Their Binding Proteins in Glucose Homeostasis

**Liam J. Murphy**

*Departments of Internal Medicine and Physiology, University of Manitoba, Winnipeg, Manitoba, Canada*

**The insulin like growth factors (IGF-I and -II) are structurally and functionally related to insulin. While insulin is a key regulator of glucose homeostasis over the short term, emerging evidence suggests that the IGFs are involved in the longer term glucose homeostasis, possibly by modulating insulin sensitivity. Unlike insulin, the IGFs are present in most biological fluids as complexes with high affinity binding proteins, the insulin-like growth factor binding proteins (IGFBPs). The IGFBPs regulate the bioavailability of the IGFs. Of the six IGFBPs identified there is evidence from studies in transgenic mice that both IGFBP-1 and IGFBP-3 may have a role in glucose regulation.**

**Keywords** Forkhead Transcription Factors; Insulin Resistance; Insulin Response Elements; Transgenic Mice

### INTRODUCTION

The insulin-like growth factors, IGF-I and -II, share structural and functional similarities with insulin. Together with insulin, they make up a family of phylogenetically conserved molecules important in the regulation of both growth and metabolism. Inadequate nutrition is an important constraint on growth. Mechanisms have evolved to regulate partitioning of caloric intake between growth and basal metabolism necessary for survival of the individual and propagation of the species.

It is not surprising that molecules that are important in the regulation of growth are also important in the regulation of metabolism. No better example of this concept exists than the IGF system where not only the ligands, IGF-I and IGF-II, but also the receptor and the postreceptor signal transduction pathways share striking similarities with insulin, the major hormone involved in glucose and lipid metabolism, the insulin receptor, and the insulin receptor signal transduction pathways. In lower species, common ancestral molecules exist and probably function both as metabolic regulators such as insulin and growth factors such as the IGFs. In higher organisms, although there is some redundancy, the metabolic and growth-promoting functions have been assigned to separate molecules. Insulin, or insulins (in some higher species more than one insulin gene is expressed), functions predominantly as regulator of metabolism, whereas IGF-I and IGF-II function predominantly as growth regulators. In addition, growth hormone, the major modulator of IGF-I and other components of the IGF system, such as insulin-like growth factor–binding protein (IGFBP)-3 and acid-labile subunit, has insulin-like effects in adipocytes in vitro (Eriksson and Toenqvist, 1997; Ridderstale and Tornqvist, 1996) and modulates insulin sensitivity by a variety of mechanisms (Rizza et al., 1982; Takano et al., 2001).

A major difference between insulin and the IGFs is the presence of the IGFBPs that have high affinity for the IGFs. In this chapter, I will review the emerging evidence that the IGF system has a role in glucose homeostasis. In particular, I will focus on the role of the IGFBPs in modifying the hypoglycemic effects of the IGF-I and -II and the recent data from transgenic mouse models.

### THE HYPOGLYCEMIC EFFECTS OF THE IGFs

Although the IGFs have insulin-like effects on glucose homeostasis and can indeed substitute for insulin in insulinopenic animal models of diabetes (Barrett et al., 1989) and

Received 16 January 2003; accepted 6 April 2003.
Address correspondence to Liam J. Murphy, Room 843, John Buhler Research Centre, University of Manitoba, 715 McDermot Avenue, Winnipeg, Manitoba R3E 3P4, Canada. E-mail: ljmurph@cc.umanitoba.ca in diabetic patients (Laager et al., 1993; Morrow et al., 1994; Schoenle et al., 1991), they are considerably less potent than insulin in lowering blood sugars, reflecting their much lower affinity for the insulin receptor (Hintz et al., 1972; Marshall et al., 1974; Zapf et al., 1978). However, in most mammals, the IGFs are present in the plasma in concentrations almost 100-fold in excess of insulin. The presence of IGFBPs effectively reduces the free IGF plasma concentrations to levels comparable to insulin. A major problem in our understanding of the IGF system in various physiological states is the lack of a reliable assay technique that measures free IGF-I and IGF-II. Various techniques currently available, such as equilibrium dialysis and antibody capture, result in perturbations such that the measured free IGF-I and -II probably more accurately reflects "easily dissociable IGF." The latter may include a portion of the IGF bound to the binding proteins. Because the binding proteins differ in their affinities for IGF-I and -II, the "easily dissociable IGF fraction" measured in such assays may reflect some component of IGF-I or -II bound to certain binding proteins.

Administration of IGF-I in doses necessary to lower blood sugar in diabetic subjects and animals to levels comparable to those seen with insulin has slightly different effects in terms of tissue glucose uptake and inhibition of gluconeogenesis and protein catabolism (Laager et al., 1993). For example, in human subjects given IGF-I or insulin to achieve comparable glucose disappearance rates, IGF-I preferentially enhanced peripheral glucose uptake and augmented the decrease in whole body protein breakdown compared to insulin but had a less marked effect on suppression of hepatic glucose output (Laager et al., 1993; Barrett et al., 1989; Tomas et al., 1996). This may reflect the known differences in tissue distribution of insulin and IGF-I receptors, but there may also be tissue differences in postreceptor signaling pathways, local IGFBP abundance, and proteases involved in degradation of insulin or the IGFs.

Skeletal muscle has abundant IGF-I receptors, whereas adipose and hepatic tissues have fewer IGF-I receptors and are therefore less responsive to IGF-I than muscle (Jacob et al., 1989; Tomas et al., 1993). To add further confusion, there is also a variable abundance of hybrid insulin/IGF-I receptors. These hybrid receptors are more abundant in skeletal muscle and are functionally more similar to insulin receptors (Siddle et al., 1994; Pandini et al., 2002; Federici et al., 1998a, 1998b, 1999). Despite being functionally similar to insulin receptors and coupled to signal transduction pathways involved in metabolic processes rather than mitogenic processes, these receptors preferentially bind IGF-I compared to insulin. Furthermore, the relative abundance of hybrid receptors varies in different physiological states. Hyperinsulinemia seen in patients with type 2 diabetes and patients with insulinomas is associated with a down-regulation of insulin receptors and an increase in hybrid receptor abundance (Federici et al., 1998a, 1998b).

IGF-I has proven to be therapeutically useful in treating diabetic patients with severe insulin resistance (Cheetham et al., 1994; Morrow et al., 1994; Schoenle et al., 1991). This effect may be due to its action on the IGF-I receptor, a preferential effect of IGF-I on IGF-I/insulin hybrid receptors, or possibly via sensitization of post–insulin receptor signal transduction pathways. A similar insulin-sensitizing effect of IGF-I in streptozotocin-treated rats has been observed, although the mechanisms responsible have not been elucidated (Tomas et al., 1993).

### THE ROLE OF THE IGFBPs IN GLUCOSE HOMEOSTASIS

Six IGFBPs have been identified and characterized and isoforms of a number of these are present in various biological fluids. These isoforms result from post-translation modification such as *N*-glycosylation for IGFBP-3 and -4 (Wood et al., 1988; Ceda et al., 1991), *O*-glycosylation for IGFBP-6 (Bach et al., 1992), and phosphorylation for IGFBP-1, -3, and -5 (Elgin et al., 1987; Frost and Tseng, 1991; Hoeck and Mukku, 1994; Coverley and Baxter, 1997). Overall amino acid sequence similarity between the IGFBPs varies from 47% to 60%, with IGFBP-3 and -5 being the most closely related. Similarity is greatest at the amino and carboxyl ends of the binding proteins, the regions of these proteins that are involved in formation of the high-affinity IGF binding site. The mid region of the IGFBPs shows the most sequence variation (Shimasaki and Ling, 1991; Rechler, 1993). The molecular pocket for high-affinity binding of IGF-I is generated by both the amino- and carboxyl-terminal ends, which are kept in close proximity to one another by the secondary structure of the IGFBPs, dictated by the highly conserved cysteine residues.

The functional role of the binding proteins in glucose homeostasis is unclear. A simplistic view is that they serve to block the insulin-like activity of the relatively large concentrations of the IGFs present in the circulation yet facilitate delivery of the IGFs to tissues to exert the anabolic effects of IGFs (Baxter, 1994). Indeed, the discovery of the IGFBPs dates back to the use of the rat fat pad bioassay to measure nonsuppressible insulin-like activity in plasma (Burgi et al., 1966). The fortuitous observation that activity of plasma samples in this assay could be enhanced by acid-ethanol extraction was the first clue to the existence of the binding proteins. A number of investigators have been able to demonstrate that the IGFBPs are able to inhibit the biological actions of the IGFs in various bioassays, including those in which glucose uptake was measured (Meuli et al., 1978; Knauer and Smith, 1980; Burch et al., 1990; Ritvos et al., 1988). For example, IGFBP-3 is able to inhibit the IGF-I effect on lipogenesis and glucose oxidation in porcine adipose tissue (Walton et al., 1989).

Several studies suggest that the effects of the binding proteins on IGF-I action may not be simple inhibition. Combinations of IGF and IGFBP-3 are more potent in a variety of in vitro and in vivo assay systems than IGF-I alone (DeMellow and Baxter, 1988; Bagi et al., 1994, 1995; Clemmons et al., 2000). The binding proteins themselves appear to be able to interact with cell surface proteins and may facilitate delivery of the IGFs to target cells (DeVroede et al., 1986), and this may explain the in vitro enhancement of IGF-I action, whereas prolongation of biological half-life of IGF-I and differential tissue uptake of IGF-I may account for the enhanced biological potency of the IGFBP-3/IGF-I complex in vivo (Bagi et al., 1994, 1995). Similarly, nonphosphorylated IGFBP-1 can enhance the activity of IGF-I under certain circumstances, at least in vitro (Elgin et al., 1987).

The majority of the insulin-like activity present in serum is due to the IGFs rather than insulin itself (Burgi et al., 1966). Because most of the IGF-I and -II present in the circulation is bound to the IGFBPs, predominantly IGFBP-3, insulin is able to acutely regulate the blood glucose. Notwithstanding this, free, unbound IGF-I and -II, which constitute a small proportion of the total IGFs present in plasma (Frystyk et al., 1999), may have some role in short-term glucose regulation. Several types of data support this hypothesis. These include the acute changes in IGFBP-1 expression that accompany food intake (Murphy et al., 1991), the hyperglycemia that accompanies infusion of IGFBP-1 (Lewitt et al., 1991) and IGFBP-3 (Vuguin et al., 2001), the glucose intolerance observed in liver-specific IGF-I knockout mice (Yakar et al., 2001), and the hyperglycemia observed in Tg mice overexpressing IGFBP-1 (Rajkumar et al., 1995) and IGFBP-3 (Silha et al., 2002).

Of the six IGFBPs, there is convincing evidence that IGFBP-1 and -3 are involved in glucose homeostasis. As yet there are no reports that directly address the role of the other binding proteins in glucose homeostasis.

### IGFBP-1 and Glucose Homeostasis

Although this binding protein probably constitutes a relatively small proportion of the total IGF-binding capacity of serum from adult rodents and human subjects, its abundance in the plasma and its expression in various tissues is acutely regulated in response to nutritional deprivation and is tightly regulated by insulin (Suikkari et al., 1988, 1989). These observations following the developments of a reliable radioimmunoassay and molecular probes for IGFBP-1 provided the initial clue that IGFBP-1 may have a role in regulating metabolic homeostasis.

IGFBP-1 has a short half-life in the circulation (Lewitt et al., 1991), possibly due to the fact that, unlike some other IGFBPs, it is not glycosylated. In addition, IGFBP-1 has a Pro-Glu-Ser-Thr sequence that is present in many proteins that have a rapid turnover (Julkunen et al., 1988). IGFBP-1 appears to be present in plasma and other biological fluids in multiple isoforms and a least one form of human IGFBP-1 can actually enhance the action of IGF-I (Elgin et al., 1987). The isoforms of IGFBP-1 appear to be the result of phosphorylation (Frost and Tseng, 1991) although other post-translational modifications have not been extensively investigated. IGFBP-1 undergoes serine phosphorylation that enhances its affinity for IGF-I by six- to eight-fold. Phosphorylation of IGFBP-1 also enhances its ability to inhibit IGF-I actions, including the hypoglycemic effects of IGF-I (Sakai et al., 2001).

In both human subjects and rodents, plasma IGFBP-1 concentrations appear to be inversely correlated with serum insulin levels (Murphy et al., 1991; Suikkari et al., 1988) and elevated IGFBP-1 concentrations are apparent in normal individuals after an overnight fast (Baxter and Cowell, 1987; Busby et al., 1988). Insulin markedly suppresses IGFBP-1 synthesis in liver explants (Lewitt and Baxter, 1989) and this can be explained by a suppressive effect of insulin on IGFBP-1 gene transcription. This inhibition of IGFBP-1 expression is mediated via an insulin response element (IRE) (Unterman et al., 1992; Suwanichkul et al., 1993; Robertson et al., 1994). The IRE motif, CAAAA(C/T)AA, is similar to that found in other insulin-inhibited genes and interacts with forkhead type transcription factor, FKHR (Rena et al., 1999), the homologue of the forkhead/winged-helix transcription factor *daf-16* identified in *Caenorhabditas elegans* (Ogg et al., 1997). Insulin, and probably IGF-I also, working via activation of the phosphatidylinositol 3-kinase pathway, leads to protein kinase B/Akt-mediated phosphorylation of FKHR. In its nonphosphorylated form, FKHR interacts with the IRE and *trans*-activates IGFBP-1 expression (Durhan et al., 1999). As a consequence of this serine phosphorylation, there is nuclear export of FKHR and disrupted *trans*-activation of IGFBP-1 and other insulin-regulated genes (Guo et al., 1999). Cotransfection of antisense FKHR also inhibits IGFBP-1 transcription, indicating that the interaction of the nonphosphorylated FKHR with the IRE is responsible for basal IGFBP-1 transcription. In hepatocytes with abundant insulin receptors and sparse IGF-I receptors, insulin is probably the major regulator of FKHR phosphorylation, whereas in other cell types where IGF-I receptors are abundant, IGF-I may be more important in regulating FKHR phosphorylation (Rena et al., 1999). This is of potential relevance because FKHR phosphorylation is important not only in the expression of IGFBP-1, whose expression is largely restricted to the liver, but also in regulating the expression of a variety of genes involved in glucose homeostasis. Furthermore in the nematode, *daf-16* is critically important in regulating the changes in metabolism that accompany the quiescent phase of the nematode life cycle (Ogg et al., 1997).

In addition to reduced circulating IGF-I concentrations, it has been long appreciated that plasma from diabetic animals also contains factors that are able to inhibit IGF action in cartilage bioassays (Phillips et al., 1979). Although the nature and functional role of these inhibitors have never been fully clarified, it appears that part, or possibly most, of this inhibitory activity is due to enhanced IGFBP concentrations, in particular IGFBP-1. An increased concentration of IGFBP-1 in sera from diabetic rats was initially reported by Unterman's group (Unterman et al., 1992) and subsequent studies demonstrated an increase in IGFBP-1 mRNA expression in hepatic and renal tissues from streptozotocin diabetic rats (Luo and Murphy, 1991). Administration of insulin to diabetic rats resulted in a decrease in IGFBP-1 gene transcription, and IGFBP-1 in the circulation.

The regulation of IGFBP-1 in relationship to nutrition suggested that this binding protein is involved in regulating glucose homeostasis. Initially, studies with infused human plasma-derived IGFBP-1 in rats demonstrated only a modest hyperglycemic effect. However, this initial observation was convincingly confirmed using transgenic mouse technology. Several groups have now generated transgenic mice overexpressing IGFBP-1 (Dai et al., 1994; Rajkumar et al., 1995; Gay et al., 1997; Crossey et al., 2000). In most (Rajkumar et al., 1995; Crossey et al., 2000) but not all (Dai et al., 1994) reports, mild glucose intolerance and/or hyperglycemia were reported in the IGFBP-1 transgenic mice. In addition, Rajkumar and colleagues (1996b) demonstrated that the hypoglycemic effect of IGF-I, but not that of des (1–3) IGF-I, an analog with little affinity for IGFBP-1, was markedly attenuated in IGFBP-1 transgenic mice (Figures 1 and 2).

In addition to the levels and tissue distribution of expression of the transgene, the phosphorylation status of the IGFBP-1 transgene appears to be important in the modulation of glucose homeostasis. This was clearly illustrated by comparison of transgenic mice that express human IGFBP-1 or rat IGFBP-1 transgenes (Sakai et al., 2001). Both strains of mice expressed high concentrations of IGFBP-1 in serum and tissues. However, human IGFBP-1 transgenic mice did not show glucose intolerance and exhibited no significant intrauterine growth retardation (Dai et al., 1994), whereas rat IGFBP-1 transgenic mice showed fasting hyperglycemia and intrauterine growth restriction (Rajkumar et al., 1996a). The explanation for this difference appears to be related in part to the phosphorylation state of the transgene product. Only half of the IGFBP-1 in serum from the human IGFBP-1 transgenic mice was phosphorylated, whereas most of the IGFBP-1 in the serum of the mice expressing the rat IGFBP-1 was phosphorylated (Sakai et al., 2001). This difference may be due to relative inability of the mouse IGFBP-1 kinase to phosphorylate human IGFBP-1 compared to rat IGFBP-1. Kinase activity purified from mouse liver cells phosphorylate mouse and rat IGFBP-1 in vitro, but it did not phosphorylated human IGFBP-1 (Sakai et al., 2001). Clearly more than one kinase is involved in phosphorylation of IGFBP-1 because some phosphorylated IGFBP-1 transgene product was present in the plasma from human IGFBP-1 transgenic mice (Dai et al., 1994). Furthermore, using a different human IGFBP-1 transgene construct, Crossey and colleagues (2000) were able to demonstrate hyperinsulinemia and glucose intolerance in their transgenic mice.

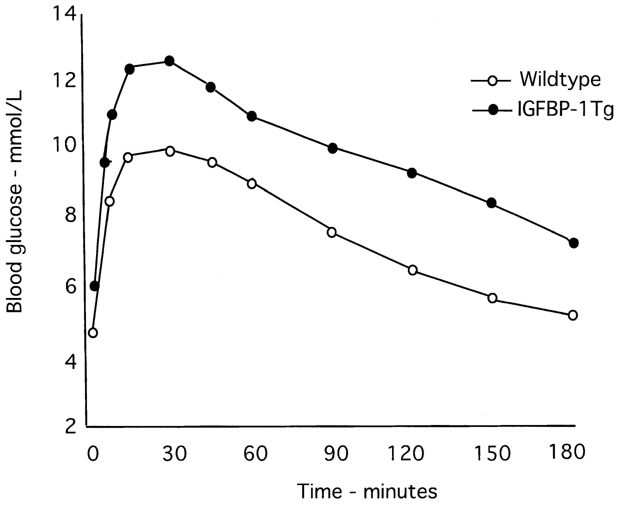

**FIGURE 1**
Glucose intolerance in IGFBP-1 transgenic mice. The plasma glucose response to an intraperitoneal glucose challenge. (Reproduced with permission from Rajkumar et al., 1996b.)

On a molar basis, the IGFs are present in the circulation in a 100-fold excess compared to insulin. Although the IGFs have only about 5% of the insulin-like activity of insulin, they represent the vast majority of the extractable insulin-like activity present in mammalian serum. Only a small percentage of the circulating IGF-I is unbound. The actual percentage of the total circulating IGF-I that is able to interact with the insulin receptor and exert the hypoglycemic effect is unclear. The data from IGFBP-1 transgenic mouse models are consistent with the hypothesis that the IGFs do indeed have some role in glucose homeostasis. However, this may be a rather simplistic explanation for the glucose intolerance observed in IGFBP-1 transgenic mice. Although glucose-intolerant IGFBP-1 transgenic mice are characterized by hyperinsulinemia (Rajkumar et al., 1996a; Crossey et al., 2000) and an increased pancreatic islet

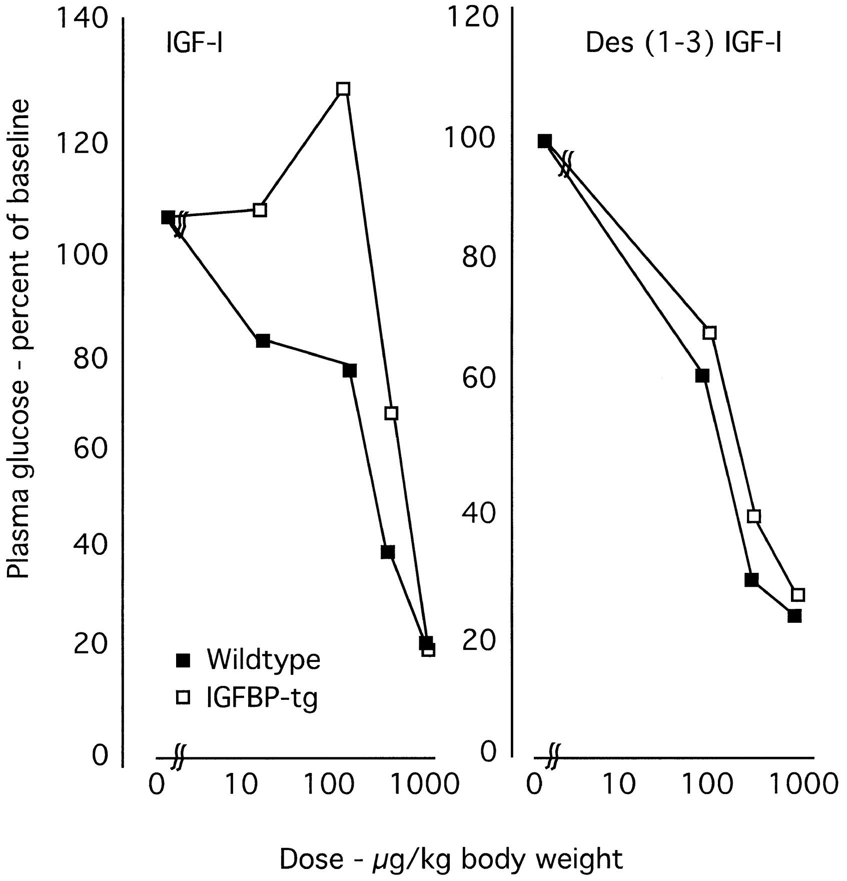

**FIGURE 2**
The hypoglycemic effects of IGF-I and des (1–3) IGF-I in wild-type and IGFBP-1 transgenic mice. (Modified from Rajkumar et al., 1996b.)

size, number, and insulin content (Dheen et al., 1996), there is also evidence of liver (Rajkumar and Murphy, 1999), adipose tissue, and skeletal muscle insulin resistance (Rajkumar et al., 1996b, 1999). The skeletal muscle insulin resistance was demonstrable both as impaired glucose uptake in isolated soleus muscle in response to insulin and also as elevated plasma amino acids concentrations, a consequence of protein catabolism in muscle (Figure 3). The skeletal muscle insulin resistance may result from a disturbance in the relative ratio of IGF/insulin hybrid receptors to insulin receptors. Reduced local IGF-I concentrations would reduce IGF-I receptor turnover, increase the abundance of IGF-I receptor subunits, and favor formation of hybrid receptors rather than insulin receptor heterodimers. Hybrid receptors are functionally more like IGF-I receptors than insulin receptors and their ability to transduce the metabolic effects of insulin are reduced (Siddle et al., 1994). However, the lack of suitable reagents to measure hybrid receptor abundance in rodents precludes directly testing this hypothesis.

In IGFBP-1 transgenic mice, pancreatic insulin content was initially increased but declined with age and this decline was accompanied by the gradual onset of hyperglycemia (Rajkumar et al., 1996b), a phenomenon reminiscent of pancreatic exhaustion observed in type II diabetes. Whether this represents an inherent pancreatic defect in IGFBP-1 transgenic mice, glucose toxicity, or a combination of both is unclear.

IGFBP-1 transgenic mice are characterized by intrauterine growth retardation (Kabada et al., 1994; Crossey et al., 2002) and abnormal placental morphology (Crossey et al., 2002). In both rodent models and human studies, intrauterine growth retardation has been associated with glucose intolerance, insulin resistance, and the development of type II diabetes later in life (Simmons et al., 2001; Yajnik, 2000). Furthermore, the IGFs, particularly IGF-II, are important in early pancreatic islet development (Petrik et al., 1999; Kido et al., 2002). Thus it is possible that the intrauterine growth retardation that accompanies IGFBP-1 overexpression may have long-lasting effects on glucose homeostasis, both in terms of tissue insulin resistance and pancreatic insulin secretory capacity. In favor of this hypothesis is the observation that glucose intolerance was demonstrable in IGFBP-1 transgenic mice where the transgene

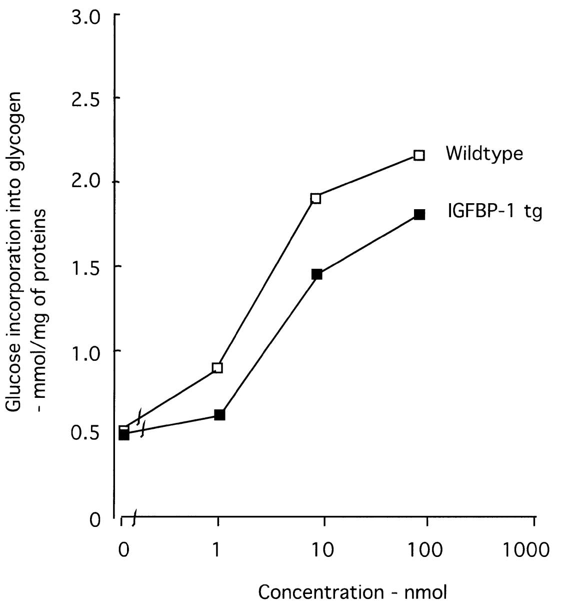

**FIGURE 3**
The effect of insulin on glucose uptake into isolated soleus muscle from wild-type and IGFBP-1 transgenic mice. (Reproduced with permission from Rajkumar et al., 1996b.)

was expressed in utero (Rajkumar et al., 1995; Crossey et al., 2002) but not in IGFBP-1 transgenic mice where postnatal induction of the transgene expression was required (Dai et al., 1994).

The IGFBP-1 transgenic mice demonstrated only modest fasting hyperglycemia. However, glycemic excursions after intraperitoneal glucose were markedly increased compared to wild-type mice (Figure 1) (Rajkumar et al., 1995). Glucose administration to fasting mice with the consequent insulin release would normally result in suppression of IGFBP-1 expression (Murphy et al., 1991b). This reduction in IGFBP-1 levels following a glucose load in wild-type mice would enhance the hypoglycemic effect of IGF-I, possibly by increasing the free, unbound IGF-I concentration. In IGFBP-1 transgenic mice where the expression of IGFBP-1 is constitutively up-regulated, this compensatory mechanism would not occur.

Null-mutant IGFBP-1 mice have been generated and appear normal according to preliminary reports (Pintar, 2001). Detailed examination of glucose homeostasis in these mice has not as yet been reported.

In summary, reports from various investigators provide clear evidence for a role for IGFBP-1 in glucose homeostasis. IGFBP-1 transgenic mice have many of the characteristic features of type II diabetes mellitus. These include a period of hyperinsulinemic normoglycemia and insulin resistance preceding fasting hyperglycemia (Rajkumar et al., 1996b).

### IGFBP-2 and Glucose Homeostasis

Unlike IGFBP-I levels, plasma IGFBP-2 concentrations do not show the same type of regulation as observed in response to fasting and refeeding. IGFBP-2 levels are not increased after an overnight fast and are not suppressed by glucose infusion in fasting human subjects (Clemmons et al., 1991). After a prolonged fast in human subjects, only a slight increase in IGFBP-2 levels was observed (Busby et al., 1988; Clemmons et al., 1991). In rodents, both fasting and streptozotocin-induced diabetes markedly increase hepatic IGFBP-2 mRNA levels but have little effect on serum IGFBP-2 levels (Rechler, 1993), probably because of the long half-life of IGFBP-2 in the circulation (Rechler, 1993).

Transgenic mice that overexpress IGFBP-2 have been generated (Hoeflich et al., 1999). These mice do not appear to display any gross abnormality in glucose homeostasis (Schneider et al., 2000). IGFBP-2 null-mutant mice have also been generated. These mice also do not appear to have any significant abnormality in glucose homeostasis (Wood et al., 1993).

### IGFBP-3 and Glucose Homeostasis

IGFBP-3 is by far the most abundant binding protein in the circulation (Jones and Clemmons, 1995). Most of the IGF-I and -II present in the circulation is bound to IGFBP-3. Levels of IGFBP-3 are relatively stable because of the long half-life of this binding protein. There is no apparent variations in IGFBP-3 levels in response to food. Both glycosylation and the ability to complex with other circulating proteins (Collett-Solberg et al., 1998), such as the acid-labile subunit (ALS) and fibronectin (Gui and Murphy, 2001), probably contribute to the long half-live of IGFBP-3. The majority of IGFBP-3 in the circulation is present as a ternary complex consisting of ALS, IGFBP-3, and IGF-I or -II.

In addition to its ability to modulate IGF availability, IGFBP-3 has been shown to have IGF-independent effects (Oh et al., 1993, 1995; Valentinis et al., 1995). The IGF-independent effects that have so far been demonstrated include effects on cellular proliferation and apoptosis. These effects may be mediated either via cell-surface binding proteins or nuclear binding sites (Oh et al., 1993; Schedlich et al., 2000). Because IGFBP-3 binds to, and colocalizes with, retinoid $\times$ receptor (R $\times$ R)-$\alpha$ in the nucleus (Liu et al., 2000), a transcription factor that is important in glucose homeostasis, the potential exists for IGFBP-3 to have both IGF-dependent and IGF-independent effects on glucose homeostasis. R $\times$ R-$\alpha$ is an important binding partner for the peroxisome proliferator-activated receptor-$\gamma$ (PPAR$\gamma$), a nuclear protein that is involved in transcriptional regulation of a variety of enzymes involved in glucose and lipid metabolism (Juge-Aubry et al., 1997).

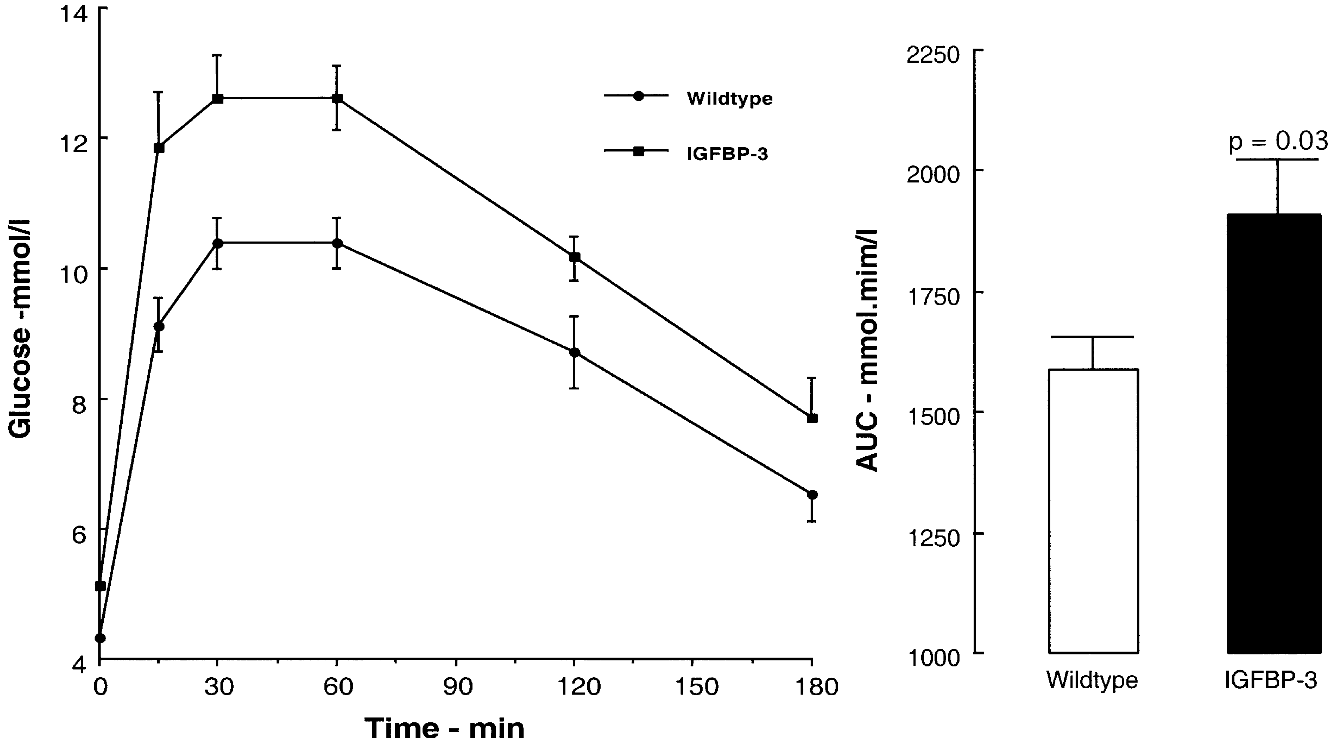

**FIGURE 4**
Plasma glucose response to an intraperitoneal glucose challenge in wild-type and IGFBP-3 transgenic mice. The plasma glucose excusions and the area under the glucose curve is shown. (Reproduced from Silha et al., 2002.)

Transgenic mice that overexpress IGFBP-3 at high levels demonstrate disturbed glucose homeostasis (Silha et al., 2002). In two different transgenic strains generated with different transgene constructs, impaired glucose tolerance and reduced insulin sensitivity were demonstrated (Figure 4). In both of these transgenic strains, there was a significant increase in the circulating concentrations of IGFBP-3, IGF-I, and their ternary complex (Modric et al., 2001). Total circulating IGF-I, the majority of which was present as ternary complex, was increased ~1.5-fold compared to wild-type mice.

In both strains of IGFBP-3 transgenic mice, the hypoglycemic response to insulin was attenuated, although this was more marked in the transgenic mouse strain where skeletal muscle expression of the IGFBP-3 transgene was greatest. In contrast, the hypoglycemic response to IGF-I was equally reduced in both transgenic mouse strains. Both strains of IGFBP-3 transgenic mice had similar levels of IGFBP-3 in the circulation; however, the CMVBP-3 transgenic mice expressed the transgene at higher levels in skeletal muscle, underlying the importance of skeletal muscle in overall insulin sensitivity (Silha et al., 2002). As discussed above, it has been suggested that IGF-I sensitizes tissues, particularly muscle, to the effects of insulin (Tomas et al., 1993). Local expression of the IGFBP-3 transgene in muscle may negate this effect.

Because circulating free IGF-I levels, and the ratio of free to total IGF-I, were similar in both IGFBP-3 transgenic mouse strains, these parameters, in contrast to skeletal muscle transgene expression, do not appear to correlate with the insulin resistance. It is likely that the measurement of these parameters in the circulation does not reflect free IGF-I levels in skeletal muscle or other tissue beds important in insulin-sensitive glucose uptake where abnormal production of IGFBP-3 has been generated by transgene expression.

The nuclear receptor R $\times$ R-$\alpha$ has been identified as a binding partner for IGFBP-3 in a yeast two-hybrid screen (Liu et al., 2000). This observation, together with the previous reports of nuclear localization of IGFBP-3 (Jacques et al., 1997; Schedlich et al., 2000), indicates that IGFBP-3 may have a role in modulating nuclear transcription of various genes involved in growth and metabolism. PPAR-$\gamma$ is also a binding partner for R $\times$ R-$\alpha$ (Juge-Aubry et al., 1997). PPAR-$\gamma$ is involved in the regulation of genes that control differentiation of preadipocytes (Morrison and Farmer, 2000) and insulin sensitivity (Lehmann et al., 1995; Steppan et al., 2001). Expression of R $\times$ R-$\alpha$ and PPAR-$\gamma$ mRNAs were similar in adipose tissue from IGFBP-3 transgenic and wild-type mice (Silha et al., 2002). Resistin mRNA, which encodes an adipokine whose expression is suppressed by PPAR-$\gamma$ agonists (Steppan et al., 2001), was also similar in IGFBP-3 transgenic and wild-type mice (Silha et al., 2002). Thus there was little evidence that the glucose intolerance of the IGFBP-3 transgenic mice was in any way related to the interaction of IGFBP-3 with the R $\times$ R-$\alpha$. If overexpression of IGFBP-3 in adipose tissue leads to increased interaction of IGFBP-3 with R $\times$ R-$\alpha$ it does not appear to result in a disturbance in expression of R $\times$ R-$\alpha$ its binding partner PPAR-$\gamma$, or one of the known downstream effectors, resistin (Silha et al., 2002). More likely the effect of overexpression of IGFBP-3 on glucose homeostasis in transgenic mice involves mechanisms similar to those that result in glucose intolerance in the IGFBP-1 transgenic mice.

The data generated from the IGFBP-3 transgenic mice clearly demonstrate that IGFBP-3 has a role in glucose homeostasis, possibly by regulating free IGF-I levels in tissues. The effects of IGFBP-3 overexpression cannot be explained by either disturbances in growth hormone secretion, adiposity, or circulating "free" IGF-I levels, because the two strains demonstrated differences in adiposity, growth hormone levels, and free IGF-I levels yet both strains demonstrated disturbances in glucose homeostasis (Silha et al., 2002). Local expression of the transgene in the tissues, particularly in skeletal muscle, appears to be more important than perturbations in circulating levels of IGF, free IGF, or growth hormone. High local levels of IGFBP-3 may reduce the free IGF in the tissue and thereby decrease the availability of IGF to the IGF-I, insulin receptor, or hybrid IGF-I/insulin receptors. It is also possible that IGFBP-3 could have direct, IGF-independent effects on glucose homeostasis, but experiments to test this hypothesis have yet to undertaken.

IGFBP-3 null-mutant mice have been generated (Pintar, 2001). Preliminary reports suggest that these mice appear to be phenotypically normal, although as yet there has been no detailed investigation of glucose homeostasis (J. Pintar, personal communications).

### FUTURE DIRECTIONS AND UNANSWERED QUESTIONS

Glucose homeostasis requires a complex interaction of many factors, only some of which are currently understood. In addition to the minute-to-minute modulation of blood sugar, glucose homeostasis involves both short- and longer-term modulation of insulin sensitivity and also partition of nutrients between different organs and tissues. Short-term modulation of insulin sensitivity would include diurnal variation in insulin sensitivity and variations that occur in relation to fasting and refeeding. Longer-term modulation of insulin sensitivity relates to changes in insulin resistance that accompany pubertal development and senescence. Circumstantial evidence suggests that the IGF system is involved in both short- and long-term modulation of insulin sensitivity. The binding proteins, by limiting the availability of IGF to interact with the IGF-I receptor or hybrid receptors, appear to have a role in regulating glucose homeostasis. The experimental observations reported to date clearly establish that IGF-I has a role in glucose homeostasis. However, the exact molecular mechanisms involved remain unresolved. The experiments with the IGFBPs, including those in which transgenic and knockout mutant models have been utilized, are relatively imprecise and have generated many more questions than answers concerning the exact role of the IGFs in glucose homeostasis. The IGFBP knockout mouse models have as yet yielded little information, possibly because of the redundancy in the binding proteins or because the appropriate experiments have yet be done.

The overexpression of IGFBP-1 and IGFBP-3 in transgenic mice has demonstrated the potential role of the IGFs in glucose homeostasis but has also raised questions that can only be addressed with more sophisticated experiments. For example, what effects would the local overexpression of IGFBP-3 or IGFBP-1 in skeletal muscle, adipose tissue, or certain cells in the brain or pancreas have on glucose homeostasis? Furthermore, the overexpression of both these binding proteins results in some degree of intrauterine growth retardation, which may have effects of resetting glucose homeostatic mechanisms. Additional experiments with tissue-specific overexpression and overexpression that can be reliably turned on and off at various stages in intrauterine and postnatal development may well provide further insight into this area. The regulation of post-translational modification of the binding proteins has received little attention. Phosphorylation and dephosphyorylation of IGFBP-1 have profound effects on the ability of IGFBP-1 to modulate the hypoglycemic effects of IGF-I. These processes are likely to be regulated and may have a role in modulating glucose homeostasis. Similarly, further experiments are required to understand the regulation and role of phosphorylation and glycosylation of IGFBP-3.

### REFERENCES

Bach, L. A., Thotakura, N. R., and Rechler, M. M. (1992) Human insulin-like growth factor binding protein-6 is *O*-glycosylated. *Biochem. Biophys. Res. Commun.,* **186,** 301–307.

Bagi, C. M., Brommage, R., Deleon, L., Adams, S., Rosen, D., and Sommer, A. (1994) Benefit of systemically administered rhIGF-I/IGFBP-3 on cancellous bone in ovariectomized rats. *J. Bone Miner. Res.,* **9,** 1301–1312.

Bagi, C. M., van der Meulen, M., Brommage, R., Rosen, D., and Summer, A. (1995) The effect of systemically administered rhIGF-I/IGFBP complex on cortical bone strength and structure in ovarectomized rats. *Bone,* **16,** 559–565.

Barett, J. R., Plewe, G., Fagin, K. D., and Sherwin, R. S. (1989) Acute effects of insulin-like growth factor I on glucose and amino acid metabolism in the awake fasted rat. Comparison with insulin. *J. Clin. Invest.,* **83,** 1717–1723.

Baxter, R. C. (1994) Insulin-like growth factor binding proteins in the human circulation: A review. *Horm. Res.,* **42,** 140–144.

Baxter, R. C., and Cowell, C. T. (1987) Diurnal rhythm of growth hormone-independent binding protein for insulin-like growth factors in human plasma. *J. Clin. Endocrinol. Metab.,* **65,** 432–439.

Burch, W. M., Correa, J., Shively, J. E., and Powell, D. R. (1990) The 25 kilodalton insulin-like growth factor (IGF)-binding protein inhibits both basal and IGF-I mediated growth of chick embryo pelvic cartilage in vitro. *J. Clin. Endocrinol. Metab.,* **70,** 173–179.

Burgi, H., Muller, W. A., Humbel, R. E., Labhart, A., and Froesch, E. R. (1996) Non-suppressible insulin-like activity of human serum. I. Physiochemical properties, extraction and partial purification. *Biochem. Biophys. Acta,* **121,** 349–359.

Busby, W. H., Snyder, D. K., and Clemmons, D. R. (1988) Radioimmunoassay of a 26,000-dalton plasma insulin-like growth factor-binding protein: Control by nutritional variables. *J. Clin. Endocrinol. Metab.,* **67,** 1225–1230.

Ceda, G. P., Fielder, P. J., Henzel, W. J., Louie, A., Donovan, S. M., Hiffman, A. R., and Rosenfeld, R. G. (1991) Differential effects of insulin-like growth factor (IGF)-I and IGF-II on the expression of IGF binding proteins (IGFBPs) in a rat neuroblastoma cell line: Isolation and characterization of two forms of IGFBP-4. *Endocrinology,* **128,** 2815–2824.

Cheetham, T. D., Taylor, A., Holly, J. M. P., Clayton, K., Cwyfan-Hughes, S., and Dunger, D. B. (1994) The effects of human insulin-like growth factor-I (IGF-I) administration on the levels of IGF-I, IGF-II and IGF-binding proteins in adolescents with insulin-dependent diabetes mellitus. *J. Endocrinol.,* **142,** 367–374.

Clemmons, D. R., Moses, A. C., McKay, M. J., Sommer, A., Rosen, D. M., and Ruckle, J. (2000) The combination of insulin-like growth factor I and insulin-like growth factor-binding protein-3 reduces insulin requirements in insulin-dependent type 1 diabetes: Evidence for in vivo biological activity. *J. Clin. Endocrinol. Metab.,* **85,** 1518–1524.

Clemmons, D. R., Snyder, D. K., and Busby, W. H. (1991) Variables controlling the secretion of insulin-like growth factor binding protein 2 in normal human subjects. *J. Clin. Endocrinol. Metab.,* **73,** 727–733.

Collett-Solberg, P. F., Nunn, S. E., Gibson, T. B., and Cohen, P. (1998) Identification of novel high molecular weight insulin-like growth factor binding protein-3 associations in human serum. *J. Clin. Endocrinol. Metab.,* **83,** 2843–2848.

Coverley, J. A., and Baxter, R. C. (1997) Phosphorylation of insulin-like growth factor binding proteins. *Mol. Cell Endocrinol.,* **128,** 1–5.

Crossey, P. A., Jones, J. S., and Miell, J. P. (2000) Dysregulation of the insulin/IGF binding protein-1 axis in transgenic mice is associated with hyperinsulinemia and glucose intolerance. *Diabetes,* **49,** 457–465.

Crossey, P. A., Pillai, C. C., and Miell, J. P. (2002) Altered placental development and intrauterine growth restriction in IGF binding protein-1 transgenic mice. *J. Clin. Invest.,* **110,** 411–418.

Dai, Z., Xing, Y., Boney, C. M., Clemmons, D. R., and D'Ercole, A. J. (1994) Human insulin-like growth factor-binding protein-1 (hIGFBP-1) in transgenic mice: Characterization and insights into the regulation of IGFBP-1 expression. *Endocrinology,* **135,** 1316–1327.

DeMellow, J. S. M., and Baxter, R. C. (1988) Growth hormone dependent insulin like growth factor (IGF) binding protein both inhibits and potentiates IGF-I stimulated DNA synthesis in human fibroblasts. *Biochem. Biophys. Res. Commun.,* **156,** 199–204.

DeVroede, M. A., Tseng, L. Y., Katsoyannis, P. G., Nissley, S. P., and Rechler, M. M. (1986) Modulation of insulinlike growth factor-I binding to human fibroblasts monolayer cultures by insulin like growth factor carrier proteins released into the incubation medium. *J. Clin. Invest.,* **77,** 602–609.

Dheen, S. T., Rajkumar, K., and Murphy, L. J. (1996) The effect of insulin-like growth factors (IGF) on pancreatic islet function in IGF binding protein-1 transgenic mice. *Diabetologia,* **39,** 1249–1254.

Durham, S. K., Suswanichkul, A., Scheimann, A. O., Yee, D., Jackson, J., Barr, F. G., and Powell, D. R. (1999) KFHR binds the insulin response element in the insulin-like growth factor binding protein-1 promoter. *Endocrinology,* **140,** 3140–3146.

Elgin, R. G., Busby, W. H., and Clemmons, D. R. (1987) An insulin-like growth factor (IGF) binding protein enhances the biological response to IGF-I. *Proc. Natl. Acad. Sci. U. S. A.,* **84,** 3254–3258.

Eriksson, H., and Tornqvist, H. (1997) Specific inhibition of the cGMP-inhibited cAMP phosphodiesterase blocks the insulin-like antilipolytic effect of growth hormone in rat adipocytes. *Mol. Cell Biochem.,* **169,** 37–42.

Federici, M., Giaccari, A., Hribal, M. L., Giovannone, B., Lauro, D., Morviducci, L., Pastore, L., Tamburrano, G., Lauro, R., and Sesti, G. (1999) Evidence for glucose/hexosamine in vivo regulation of insulin/IGF-I hybrid receptor assembly. *Diabetes,* **48,** 2277–2285.

Federici, M., Lauro, D., D'Adamo, M., Giovannone, B., Porzio, O., Mellozzi, M., Tamburrano, G., Sbraccia, P., and Sesti, G. (1998a) Expression of insulin/IGF-I hybrid receptors is increased in skeletal muscle of patients with chronic primary hyperinsulinemia. *Diabetes,* **47,** 87–92.

Federici, M., Porzio, O., Lauro, D., Borboni, P., Giovannone, B., Zucaro, L., Hribal, M. L., and Sesti, G. (1998b) Increased abundance of insulin/insulin-like growth factor-I hybrid receptors in skeletal muscle of obese subjects is correlated with in vivo insulin sensitivity. *J. Clin. Endocrinol. Metab.,* **83,** 2911–2915.

Frost, R. A., and Tseng, L. (1991) Insulin-like growth factor-binding protein-1 is phosphorylated by cultured human endometrial stromal cells and multiple protein kinases *in vitro*. *J. Biol. Chem.,* **266,** 18082–18088.

Frystyk, J., Delhanty. P. J. D., Skjaerbaek, C., and Baxter, R. C. (1999) Changes in the circulating IGF system during short-term fasting and refeeding in rats. *Am. J. Physiol. Endocrinol. Metab.,* **277,** E245–E252.

Gay, E., Seurin, D., Babajko, S., Doublier, S., Cazillis, M., and Binoux, M. (1997) Liver-specific expression of human insulin-like growth factor binding protein-1 in transgenic mice: Repercussions on reproduction, ante- and perinatal mortality and postnatal growth. *Endocrinology,* **138,** 2937–2947.

Gui, Y., and Murphy, L. J. (2001) Insulin like growth factor (IGF)-binding protein-3 (IGFBP-3) binds to fibronectin (FN): Demonstration of IGF-I/IGFBP-3/FN ternary complexes in human plasma. *J. Clin. Endocrinol. Metab.,* **86,** 2104–2110.

Guo, S., Rena, G., Cichy, S., He, X., Cohen, P., and Unterman, T. (1999) Phosphorylation of serine 256 by protein kinase B disrupts transactivation by FKHR and mediates effects of insulin on insulin-like growth factor-binding protein-1 promoter activity through a conserved insulin response sequence. *J. Biol. Chem.,* **274,** 17184–17192.

Hintz, R. L., Clemmons, D. R., Underwood, L. E., and Van Wyk, J. J. (1972) Competitive binding of somatomedin to the insulin receptor of adipocytes, chondrocytes, and liver membranes. *Proc. Natl. Acad. Sci. U. S. A.,* **69,** 2351–2353.

Hoeck, W. G., and Mukku, V. R. (1994) Identification of the major sites of phosphorylation in IGF binding protein-3. *J. Cell Biochem.,* **56,** 262–273.

Hoeflich, W., Wu, M., Mohan, S., Foll, J., Wanke, R., Froehlich, T., Arnold, G. J., Lahm, H., Kolb, H. J., and Wolf, E. (1999) Overexpression of insulin-like growth factor binding protein-2 in transgenic mice reduces postnatal body weight gain. *Endocrinology,* **140,** 5488–5496.

Jacob, R., Barret, G., Fagin, K. D., and Sherwin, R. S. (1989) Acute effects of insulin-like growth factor I on glucose and amino acid metabolism in the awake fasted rat. *J. Clin. Invest.* **83,** 1717–1723.

Jacques, G., Noll, K., Wegmann, B., Witten, S., Kogan, E., Radulescu, R. T., and Havemann, K. (1997) Nuclear localization of insulin-like growth factor binding protein 3 in a lung cancer cell line. *Endocrinology,* **138,** 1767–1770.

Jones, J. I., and Clemmons, D. R. (1995) Insulin-like growth factors and their binding proteins: Biological actions. *Endocr. Rev.,* **16,** 3–34.

Juge-Aubry, C., Pernin, A., Favez, T., Burger, A. G., Wahli, W., Meier, C. A., and Desvergne, B. (1997) DNA binding properties of peroxisome proliferator-activated receptor subtypes on various natural peroxisome proliferator response elements. *J. Biol. Chem.,* **272,** 25252–25259.

Julkunen, M., Koistinen, R., Aalto-Setala, K., Seppala, M., Janne, O. A., and Kontula, K. (1988) Primary structure of human insulin-like growth factor-bindingprotein/placental protein 12 and tissue-specific expression of its mRNA. *FEBS Lett.,* **236,** 295–302.

Kido, Y., Nakae, J., Hribal, M. L., Xuan, S., Efstratiadis, A., and Accili, D. (2002) Effects of mutations in the insulin-like growth factor signaling system on embryonic pancreas development and beta-cell compensation to insulin resistance. *J. Biol. Chem.,* **277,** 36740–36477.

Knauer, D. J., and Smith, G. L. (1980) Inhibition of biological activity of multiplication-stimulating activity by binding to its carrier protein. *Proc. Natl. Acad. Sci. U. S. A.,* **77,** 7252–7259.

Laager, R., Ninnis, R., and Keller, U. (1993) Comparison of the effects or recombinant human Igf on glucose and leucine kinetics in humans. *J. Clin. Invest.,* **92,** 1903–1909.

Liu, B., Lee, H.-Y., Weinzimer, S. A., Powell, D. R., Clifford, J. L., Kurie, J. M., and Cohen, P. (2000) Direct functional interactions between insulin-like growth factor-binding protein-3 and retinoid × receptor—a regulate transcriptional signaling and apoptosis. *J. Biol. Chem.,* **275**, 33607–33613.

Lehmann, J. M., Moore, L. B., Smith-Oliver, T. A., Wilkison, W. O., Willson, T. M., and Kliewer, S. A. (1995) An antidiabetic thiazolidinedione is a high affinity ligand for peroxisome proliferator-activated receptor gamma (PPAR-gamma). *J. Biol. Chem.,* **270,** 12953–12956.

Lewitt, M. S., and Baxter, R. C. (1989) Regulation of growth hormone-independent insulin-like growth factor-binding protein (BP-28) in cultured human fetal liver explants. *J. Clin. Endocrinol. Metab.,* **69,** 246–251.

Lewitt, M. S., Denyer, G. S., Cooney, G. J., and Baxter, R. C. (1991) Insulin-like growth factor binding protein-1 modulates blood glucose levels. *Endocrinology,* **129,** 2254–2256.

Luo, J. M., and Murphy, L. J. (1991) Differential expression of insulin-like growth factor-I and insulin-like growth factor binding protein-1 in the diabetic rat. *Mol. Cell Biochem.,* **103,** 41–50.

Marshall, R. N., Underwood, L. E., Voina, S. J., Foushee, D. B., and Van Wyk, J. J. (1974) Characterization of the insulin and somatomedin-C receptors in human placental membranes. *J. Clin. Endocrinol. Metab.,* **39,** 2122–2125.

Modric, T., Silha, J., Shi, Z., Gui, Y., Suwanichkul, A., Durham, S. K., Powell, D. R., and Murphy, L. J. (2001) Phenotypic manifestations of insulin-like growth factor binding protein-3 overexpression in transgenic mice. *Endocrinology,* **142,** 1958–1967.

Morrison, R. F., and Farmer, S. R. (2000) Hormonal signaling and transcriptional control of adipocyte differentiation. *J. Nutr.,* **130,** 3116–3121.

Morrow, L. A., O'Brien, M. B., Moller, D. E., Flier, J. S., and Moses, A. C. (1994) Recombinant human insulin-like growth factor-I therapy improves glycemic control and insulin action in the type A syndrome of severe insulin resistance. *J. Clin. Endocrinol. Metab.,* **79,** 205–210.

Meuli, C., Zapf, J., and Froesch, E. R. (1978) NSILA-carrier protein abolishes the action of nonsuppressible insulin-like activity (NSILA-S) on perfused rat heart. *Diabetologia,* **14,** 255–261.

Murphy, L. J., Luom J., and Seneviratne, C. (1991a) Hormonal regulation of insulin-like growth factor binding protein-1 expression in the rat. *Adv. Exp. Med. Biol.,* **293,** 149–161.

Murphy, L. J., Seneviratne, C., Moreira, P., and Reid, R. (1991b) Enhanced expression insulin-like growth factor binding protein-1 in the fasted rat: The effects of insulin and growth hormone administration. *Endocrinology,* **128,** 689–696.

Ogg, S., Paradis, S., Gottlieb, S., Patterson, G. I., Lee, L., Tissenbaum, H. A., and Ruvkun, G. (1997) The forkhead transcription factor DAF-16 transduces insulin-like metabolic and longevity signals in *C. elegans. Nature,* **389,** 894–999.

Oh, Y., Gucev, Z., Ng, L., Muller, H. L., and Rosenfeld, R. G. (1995) Antiproliferative actions of insulin-like growth factor binding protein (IGFBP)-3 in human breast cancer cells. *Prog. Growth Factor Res.,* **6,** 205–212.

Oh, Y., Muller, H. L., Pham, H., and Rosenfeld, R. G. (1993) Demonstration of receptors for insulin-like growth factor binding protein-3 on Hs578T human breast cancer cells. *J. Biol. Chem.,* **268,** 26045–26048.

Pandini, G., Frasca, F., Mineo, R., Sciacca, L., Vigneri, R., and Belfiore, A. (2002) Insulin/insulin-like growth factor I hybrid receptors have different biological characteristics depending on the insulin receptor isoform involved. *J. Biol. Chem.,* **277,** 39684–39695.

Petrik, J., Pell, J. M., Arany, E., McDonald, T. J., Dean, W. L., Reik, W., and Hill, D. J. (1999) Overexpression of insulin-like growth factor-II in transgenic mice is associated with pancreatic islet cell hyperplasia. *Endocrinology,* **140,** 2353–2363.

Pintar, J. E. (2001) Single and multiple knockouts of the IGFBPs. Program of the 83rd Annual Meeting of The Endocrine Society, Denver, CO, p. 44 (Abstract S33-1).

Phillips, L. S., Belosky, D. C., Young, H. S., and Reichard, L. A. (1979) Nutrition and somatomedins VI. Somatomedin activity and somatomedin inhibitory activity in the sera from normal and diabetic rats. *Endocrinology,* **104,** 1519–1522.

Rajkumar, K., Barron, D., Lewitt, M. S., and Murphy, L. J. (1995) Growth retardation and hyperglycemia in insulin-like growth factor binding protein-1 transgenic mice. *Endocrinology,* **136,** 4029–4034.

Rajkumar, K., Dheen, S., and Murphy, L. J. (1996a) Hyperglycemia and impaired glucose tolerance in insulin-like growth factor binding protein-1 transgenic mice. *Am. J. Physiol.,* **270,** E565–E571.

Rajkumar, K., Krsek, M., Dheen, S. T., and Murphy, L. J. (1996b) Impaired glucose homeostasis in insulin-like growth factor binding protein-1 transgenic mice. *J. Clin. Invest.,* **98,** 1818–1825.

Rajkumar, K., Modric, T., and Murphy, L. J. (1999) Impaired adipogenesis in insulin-like growth factor binding protein-1 transgenic mice. *J. Endocrinol.,* **162,** 457–465.

Rajkumar, K., and Murphy, L. J. (1999) Enhanced gluconeogenesis and hepatic insulin resistance in insulin-like growth factor binding protein-1 transgenic mice. *Biochem. Biophys. Acta,* **1426,** 491–497.

Rechler, M. M. (1993) Insulin-like growth factor binding proteins. *Vit. Horm.,* **46,** 1–114.

Rena, G., Guo, S., Cichy, S. C., Unterman, T. G., and Cohen, P. (1999) Phosphorylation of the transcription factor forkhead family member FKHR by protein kinase B. *J. Biol. Chem.,* **274,** 17179–17183.

Ridderstrale, M., and Tornqvist, H. (1996) Effects of tyrosine kinase inhibitors on tyrosine phosphorylations and the insulin-like effects in response to human growth hormone in isolated rat adipocytes. *Endocrinology,* **137,** 4650–4656.

Ritvos, O., Ranta, T., Jalkanen, J., Suikkari, A.-M., Voutilainen, R., Bohn, H., and Rutanen, E. M. (1988) Insulin-like growth factor (IGF) binding protein from human decidua inhibits the binding and biological action of IGF-I in cultured choriocarcinoma cells. *Endocrinology,* **122,** 2150.

Rizza, R. A., Mandarino, L. J., and Gerich, J. E. (1982) Effects of growth hormone on insulin action in man: Mechanisms of insulin resistance, impaired suppression of glucose production, and impaired stimulation of glucose utilization. *Diabetes,* **31,** 663–669.

Robertson, D. G., Marino, E. M., Thule, P. M., Seneviratne, C. K., and Murphy, L. J. (1994) Insulin and glucocorticoids regulate IGFBP-1 expression via a common promoter region. *Biochem. Biophys. Res. Commun.,* **200,** 226–232.

Sakai, K., D'Ercole, J., Murphy, L. J., and Clemmons, D. R. (2001) Physiological differences of phosphorylation of insulin-like growth factor binding protein-1 (IGFBP-1) in IGFBP-1 transgenic mice. *Diabetes,* **50,** 32–38.

Schedlich, L. J., LePage, S. L., Firth, S. M., Briggs, L. J., Jans, D. A., and Baxter, R. C. (2000) Nuclear import of insulin-like growth factor-binding protein-3 and -5 is mediated by the importin beta subunit. *J. Biol. Chem.,* **275,** 23462–23470.

Schneider, M. R., Lahm, H., Wu, M., Hoeflich, A., and Wolf, E. (2000) Transgenic mouse models for studying the functions of insulin-like growth factor binding proteins. *FASEB J.,* **14,** 629–640.

Schoenle, E. J., Zenobi, P. D., Torresani, T., Werder, E. A., Zachmann, M., and Froesch, E. R. (1991) Recombinant insulin-like growth factor I (rIGF-I) reduces hyperglycemia in patients with extreme insulin resistance. *Diabetologia,* **34,** 675–679.

Shimasaki, S., and Ling, N. (1991) Identification and molecular characterization of insulin-like growth factor binding proteins (IGFBP-1, -2, -3, -4, -5 and -6). *Prog. Growth Factor Res.,* **3,** 243–266.

Siddle, K., Soos, M. A., Field, C. E., and Nave, B. T. (1994) Hybrid and atypical insulin/insulin-like growth factor I receptors. *Horm. Res.,* **41(Suppl 2),** 56–64.

Silha, J. V., Gui, Y., and Murphy, L. J. (2002), Impaired glucose homeostasis in insulin-like growth factor binding protein-3 transgenic mice. *Am, J. Physiol.,* **283,** E937–E945.

Simmons, R. A., Templeton, L. J., and Gertz, S. J. (2001) Intrauterine growth retardation leads to the development of type 2 diabetes in the rat. *Diabetes,* **50,** 2279–2286.

Steppan, C. M., Bailey, S. T., Bhat, S., Brown, E. J., Banerjee, R. R., Wright, C. M., Patel, H. R., Ahima, R.S., and Lazar, M. A. (2001) The hormone resistin links obesity to diabetes. *Nature,* **409,** 307–312.

Suikkari, A.-M., Koivisto, V. A., Koistinen, R., Seppala, M., and Yki-Jarvivnen, H. (1989) Dose-response characteristics for suppression of low molecular weight plasma insulin-like growth factor-binding protein by insulin. *J. Clin. Endocrinol. Metab.,* **68,** 135–140.

Suikkari, A.-M., Koivisto, V. A., Rutanen, E.-M., Yki-Jarvinen, H., Karonen, S.-L., and Seppala, M. (1988) Insulin regulates the serum levels of low molecular weight insulin-like growth factor-binding protein. *J. Clin. Endocrinol. Metab.,* **66,** 266–272.

Suwanichkul, A., Morris, S. L., and Powell, D. R. (1993) Identification of an insulin responsive element in the promoter of the human gene for insulin-like growth factor binding protein-1. *J. Biol. Chem.,* **268,** 17063–17068.

Takano, A., Haruta, T., Iwata, M., Usui, I., Uno, T., Kawahara, J., Ueno, E., Sasaoka, T., and Kobayashi, M. (2001) Growth hormone induces cellular insulin resistance by uncoupling phosphatidylinositol 3-kinase and its downstream signals in 3T3-L1 adipocytes. *Diabetes,* **8,** 1891–1900.

Tomas, F. M., Knowles, S. E., Owens, P. C., Burgoyne, J. L., Chandler, C. S., and Ballard, F. J. (1996) Conjoint IGF-I and insulin infusion shows diverse interactive effects in diabetic rats. *Diabetes,* **45,** 170–177.

Tomas, F. M., Knowles, S. E., Owens, P. C., Chandler, C. S., Francis, G. L., and Ballard, F. J. (1993) Insulin-like growth factor-I and more potent variants restore growth of diabetic rats without inducing all characteristic insulin effects. *Biochem. J.,* **291,** 781–786.

Unterman, T. G., Lacson, R. G., McGary, E., Whalen, C., Purple, C., and Goswami, R. G. (1992) Cloning of the rat insulin-like growth factor binding protein-1 gene and analysis of its 5' promoter region. *Biochem. Biophys. Res. Commun.,* **185,** 993–999.

Valentinis, B., Bhala, A., DeAngelis, T., Baserga, R., and Cohen, P. (1995) The human insulin-like growth factor (IF) binding protein-3 inhibits the growth of fibroblasts with a targeted disruption of the IGF-I receptor gene. *Mol. Endocrinol.,* **9,** 361–367.

Vuguin, P. M., Shim, M. L., Cohen, P., and Barzilai, N. (2001) IGFBP-3 induces hepatic and peripheral insulin resistance in vivo. *Diabetes,* **50(Supp 2),** A268.

Walton, P. E., Gopinath, R., and Etherton, T. D. (1989) Porcine insulin-like growth (IGF) binding protein blocks IGF-I action on porcine adipose tissue. *Proc. Soc. Exp. Biol.,* **190,** 315–321.

Wood, T. L., Rogler, L., Streck, R. D., Cerro, J., Green, B., Grewal, A., and Pintar, J. E. (1993) Targeted disruption of IGFBP-2 gene. *Growth Reg.,* **8,** 5–8.

Yajnik, C. (2000) Interactions of perturbations in intrauterine growth and growth during childhood on the risk of adult-onset disease. *Proc. Nutr. Soc.,* **59**, 257–265.

Yakar, S., Liu, J-L, Fernandez, A. M., Wu, Y., Schally, A. V., Frystyk, J., Chernausek, S. D., Mejia, W., and LeRoith, D. (2001) Liver-specific *igf-1* gene deletion leads to muscle insulin insensitivity. *Diabetes,* **50,** 1110–1118.

Zapf, J., Schoenle, E., and Froesch, E. R. (1978) Insulin-like growth factors I and II: Some biological actions and receptor binding characteristics of two purified constituents on non-suppressible insulin-like activity in human serum. *Eur. J. Biochem.,* **87,** 287–296.

